# Analysis of paternal lineages in Brazilian and African populations

**DOI:** 10.1590/S1415-47572010005000067

**Published:** 2010-09-01

**Authors:** Mónica Carvalho, Pedro Brito, Virgínia Lopes, Lisa Andrade, Mª João Anjos, Francisco Corte Real, Leonor Gusmão

**Affiliations:** 1 Forensic Genetics Service, Centre Branch, National Institute of Legal Medicine, CoimbraPortugal; 2 National Institute of Legal Medicine, CoimbraPortugal; 3 Faculty of Medicine, University of Coimbra, CoimbraPortugal; 4 Institute of Pathology and Molecular Immunology, University of Porto, PortoPortugal

**Keywords:** chromosome Y, STRs, lineages, Brazil, Africa

## Abstract

The present-day Brazilian population is a consequence of the admixture of various peoples of very different origins, namely, Amerindians, Europeans and Africans. The proportion of each genetic contribution is known to be very heterogeneous throughout the country. The aim of the present study was to compare the male lineages present in two distinct Brazilian populations, as well as to evaluate the African contribution to their male genetic substrate. Thus, two Brazilian population samples from Manaus (State of Amazon) and Ribeirão Preto (State of São Paulo) and three African samples from Guinea Bissau, Angola and Mozambique were typed for a set of nine Y chromosome specific STRs. The data were compared with those from African, Amerindian and European populations. By using Y-STR haplotype information, low genetic distances were found between the Manaus and Ribeirão Preto populations, as well as between these and others from Iberia. Likewise, no significant distances were observed between any of the African samples from Angola, Mozambique and Guinea Bissau. Highly significant Rst values were found between both Brazilian samples and all the African and Amerindian populations. The absence of a significant Sub-Saharan African male component resulting from the slave trade, and the low frequency in Amerindian ancestry Y-lineages in the Manaus and Ribeirão Preto population samples are in accordance with the accentuated gender asymmetry in admixture processes that has been systematically reported in colonial South American populations.

## Introduction

South America was already inhabited when the first European settlers arrived. The first of these, more specifically the Portuguese, landed in Brazil in 1500, in territory already occupied by Amerindians. The region was quickly colonized, with the intention to explore its natural resources. Throughout the history different people from all over the world arrived at this territory, especially in the colonial period, with a great affluence from Africa due to the slave trade, thereby initiating the admixture of Amerindians, Africans and Europeans. In the 16^th^ century, on the occasion of the arrival of the Portuguese, the native population was around 2.5 million. Between the 16^th^ and 19^th^ centuries, about 4 million Sub-Saharan African slaves arrived in Brazil (IBGE, 2000). Initially, admixture was mainly between native females and Portuguese male navigators, due to the insignificant immigration of European women (Carvalho-Silva *et al.*, 2001). The subsequent migration contributed to a constant influx of both Africans and Europeans, thereby giving rise to the present very heterogeneous population, due to the different proportions of local admixture throughout the country (Ribeiro, 1995).

In this work, we attempted to study this historic influence on the genetic background of present-day populations, by analyzing two Brazilian populations, one from Manaus (State of Amazon) and the other from Ribeirão Preto (State of São Paulo). Three African populations, from Guinea Bissau, Angola and Mozambique were also included in the study, since they represent the ancestral populations of most Africans that arrived in Brazil during the period of the slave trade. The five populations were genetically characterized for Y chromosome specific STR loci by typing 9 markers (DYS19, DYS389 I, DYS389 II, DYS390, DYS391, DYS392, DYS393 and DYS385, the latter including 2 loci), whereas the Brazilian samples were typed for five SNP markers (M2, M3, M35, M213 and SRY10831). In order to evaluate the possible male contributions to our samples, a comparison was made between our data and those already available for Brazilian, Amerindian, African and European populations.

## Materials and Methods

###  DNA samples

The Y-chromosomal minimal haploptype was defined by 9 Y- STRs (DYS19, DYS389 I, DYS389 II, DYS390, DYS391, DYS392, DYS393, DYS385) in unrelated individuals from Manaus (N = 42), Ribeirão Preto (N = 65), Guinea Bissau (N = 32), Angola (N = 48) and Mozambique (N = 36). The samples from Brazil, Guinea Bissau and Mozambique were collected in district hospitals. The Brazilian samples were composed of individuals from the cities Manaus and Ribeirão Preto. The African samples from Guinea Bissau and Mozambique consisted of individuals living in the regions of Bissau and Maputo, respectively. The samples from Angola were collected in the northern region of the country, and included individuals from the villages of N'Dalatando and Lucala, in Kwanza province, and from the province of Uíge. Blood samples were obtained with written informed consent.

###  Marker typing

For samples from Manaus and Ribeirão Preto, the minimal haplotype was typed using a *PowerPlexY PCR* Amplification Kit (Promega), with primers and amplification conditions according to manufacturer's instructions. As regards the others from Guinea Bissau, Angola and Mozambique, the STRs DYS19, DYS389 I, DYS389 II, DYS390 and DYS393 were amplified as described by Gusmão *et al.* (1999). DYS385 amplification conditions complied with the methodology, as described by Schneider *et al.* (1998), whereas multiplex amplification of DYS391, DYS392, DYS393 was according to Kloosterman *et al.* (1998). Alleles were designated according to the International Society for Forensic Genetics (ISFG) guidelines for forensic analysis using Y-STRs (Gusmão *et al.*, 2006). For defining male haplogroups in population samples from Manaus and Ribeirão Preto, five Y-chromosome SNP markers (M2, M3, M35, M213 and SRY10831) were genotyped using methods as described by Silva *et al.* (2006). Haplogroup nomenclature was according to Karafet *et al.* (2008).

###  Statistical analysis

Both haplotype diversity, according to Nei (1987), and pairwise Rst genetic distances were calculated using Arlequin v. 3.0 software Excoffier *et al.* (2005), without considering DYS385. R_ST_ genetic distances were visualized in two-dimensional space by using the Multi DimensionalScaling (MDS) method included in the StatSoft, Inc. (2007) STATISTICA data analysis software system, version 8.0.

## Results and Discussion

###  Y haplotype diversity

The haplotype results obtained for the STRs (DYS19, DYS389 I, DYS389 II, DYS390, DYS391, DYS392, DYS393 and DYS385) are provided as Supplementary Material (Tables S1 to S5). Haplotype diversity was estimated in all studied populations (Table 1). A comparison of haplotype diversity revealed high levels in Manaus and Ribeirão Preto, comparable to that observed in the African samples. Although higher diversity could be expected in African samples, the presence of male lineages of different origins in the Brazilian populations might have contributed to incrementing diversity.

In the sample from Manaus, all individuals presented different haplotypes (42 unique ones) with overall haplotype diversity of 1.000 ± 0.0052. In Ribeirão Preto, 58 different haplotypes were observed, 54 of which unique. In Ribeirão Preto, the most common haplotypes represented in more than one individual (RP1, RP5, RP6 and RP19 - Table S2) correspond to or are just a few steps apart from, the most frequent haplotype in all Iberian populations (Gusmão *et al.* 2003), which represents the core haplotype within the R1b1b2-M269 haplogroup. In Manaus, this core haplotype was encountered in only one individual (M3), whereas one or two step neighbors were found in 10 individuals (M1, M4, M10, M21, M22, M30, M37, M38, M39 and M42). Therefore, by analyzing this set of Y-STRs, it is possible to infer an important male-mediated European genetic influx in both of the Brazilian populations studied.

29 different haplotypes were observed in the Guinea Bissau population, 27 of which unique. In Angola, there were 42 different haplotypes, 36 unique. Finally, in Mozambique 32 unique haplotypes were observed from a total of 34 different ones. Four different haplotypes are shared between Angola and Mozambique, and one between Angola and Guinea Bissau. Three of these shared haplotypes match the Bantu core described by Thomas *et al.* (2000), besides one differing by only one mutation step. When searching for shared haplotypes between African and Brazilian samples, a single hit was found between Angola and Manaus (M25 = A20), this also matching the Bantu modal. Apart from this haplotype found in Manaus, a search in both Brazilian samples did not reveal the presence of any other haplotype corresponding to the Bantu core. Only one haplotype was found in Ribeirão Preto that differed by a single step from the Bantu modal. Therefore, based on Y-STR results, a significant male-mediated African genetic influx could not be expected in both of these Brazilian populations.

###  Population comparison

The population samples from Manaus, Ribeirão Preto, Guinea Bissau, Mozambique and Angola were compared with different populations (listed in Table 2) through pairwise R_ST_ genetic distance analysis. The results obtained showed no significant differences between Manaus and Ribeirão Preto (R_ST_ = 0.0002, p = 0.4030). The same was observed when comparing these two populations with other urban and/or admixed South American (codes 3, 4, 5, 6, 7 and 12 in Table 2) or Iberian (13, 14, 15 and 16 - Table 2) populations, with R_ST_ values below 0.0011 (p > 0.0059). Among other American populations, a significant differentiation was found between Manaus or Ribeirão Preto, and African descendents from Rio de Janeiro (R_ST_ > 0.0817, p = 0.0002) and South Amerindians (populations 9, 10 and 11 in Table 2), R_ST_ > 0.0997 (p = 0.0015).

Within the African group, although p-values were not significant (p > 0.01), higher R_ST_ values were observed between the Guinea Bissau, Angola and Mozambique samples (0.00998 < R_ST_ < 0.05381) than those observed between the Brazilian and European samples. Highly significant R_ST_ values were found when comparing the Manaus and Ribeirão Preto with all the African populations (R_ST_ > 0.1311, p = 0.0000).

**Figure 1:**
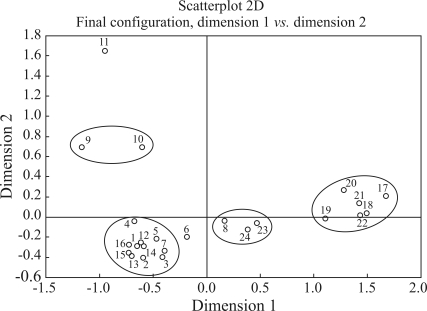
MDS plot based on population pairwise Rst values. Clusters are indicated for populations that did not significantly differ in comparison analysis based on R_ST_ p-values. Population codes are indicated in Table 2.

In multidimensional scaling (MDS) plot of pairwise R_ST_ genetic distances, based on Y-STR data (Figure 1), it is possible to note the formation of different clusters, these including a European and an African group, as well as two other clusters formed by European/Amerindian and European/African mixed populations. As expected, populations from the Guinea Bissau, Angola and Mozambique groups are on a line with other populations from continental Africa. Manaus and Ribeirão Preto clearly group with European populations from Iberia, together with other urban population samples from South America. These two populations stand well apart from the remaining clusters of African, Amerindian or admixed ancestry.

###  Y-SNP haplogroups in the Brazilian populations

Five SNPs were typed in the Manaus and Ribeirão Preto samples, in order to trace the origin of the Y-chromosomes in the current population. In each population sample, a single chromosome belonged to the most frequent Sub-Saharan African haplogroups by carrying the M2 mutation (Tables S1 and S2). In Ribeirão Preto, a second African lineage could be found, which lacks the M213 and SRY10831 mutations, therefore being classified in paragroup A*. One out of 65 samples from Ribeirão Preto and 4 out of 42 from Manaus carried both the M213 and M3 mutations that characterize Amerindian haplogroups. The remaining samples, were classified in F* (except Q3), E1b1b1 - M35 or Y* (xA, E1b1a-b1, F*).

Based on the SNP results, we concluded that Europe is the main source of paternal lineages existing in the present-day population of Ribeirão Preto (95.4%), with African and Amerindian lineages only representing 3.1% and 1.5% of the chromosomes, respectively. In Manaus, the origin of most chromosomes can also be traced to Europe (88.1%), although a higher Amerindian component was found (9.5%). Only a single African-ancestry chromosome (2.4%) was detected in the sample from Manaus.

## Conclusions

Many studies have been carried out to characterize the genetic diversity of Brazilian populations aiming to better understand colonization processes and the demographic history of its native populations (*e.g.* Bortolini *et al.* 2003; Abe-Sandes *et*. *al.* 2004; Silva *et al.* 2006). These studies systematically revealed a particularly sub-structured country, with populations from distinct regions differing in their proportion of African, Amerindian and European ancestries. Studies of mtDNA and Y-chromosome markers also revealed much higher genetic differentiation at the maternal gene-pool level than at the paternal counterpart (Marrero *et al.* 2005). Indeed, as regards Y chromosome lineage, a high European contribution was observed in most Brazilian samples. This was also evident in the present study, where Brazilian samples, as well as all other general population samples countrywide, presented much lower genetic distances when compared with Europeans than with Africans or Amerindians. A European contribution was also evident in the South Amerindian sample studied by Leite *et al.* (2008) which presented a similar distance (R_ST_ = 0.18) to the Toba Amerindian sample and any of the Iberian samples. Nevertheless, from the SNP results it can be inferred that, although genetic distance analysis based on STR profiles allowed to identify the main European contribution to the Brazilian samples, it was not able to detect minor contributions. In fact, the almost 10% Amerindian contribution to the Manaus sample was insufficient to produce significant genetic distance values between Manaus and both Ribeirão Preto or Iberian populations.

The absence of a significant Sub-Saharan African male component resulting from the slave trade or Amerindian ancestry Y-lineages, in the Manaus and Ribeirão Preto population samples, is in accordance with pronounced gender asymmetry in admixture processes that has been systematically reported in colonial South American populations.

## Supplementary Material

The following online material is available for this article:

Table S1 Y chromosome haplotype distribution in the Manaus population sample (N = 42).

Table S2 Y chromosome haplotype distribution in the Ribeirão Preto population sample (N = 65).

Table S3 Y chromosome haplotype distribution in the Guinea Bissau population sample (N = 32).

Table S4 Y chromosome haplotype distribution in the Angola population sample (N = 48).

Table S5 Y chromosome haplotype distribution in the Mozambique population sample (N = 36).

This material is available as part of the online article from http://www.scielo.br/gmb.

## Figures and Tables

**Table 1:** Number of different and unique haplotypes, and haplotype diversity in population samples from Manaus, Ribeirão Preto, Guinea Bissau, Angola and Mozambique.

Population	N	Number of different haplotypes	Number of unique haplotypes	Haplotype diversity
Manaus	42	42	42	1.0000 ± 0.0052
Ribeirão Preto	65	58	54	0.9947 ± 0.0044
Guinea Bissau	32	29	27	0.9919 ± 0.0110
Angola	48	42	36	0.9947 ± 0.0054
Mozambique	36	34	32	0.9968 ± 0.0075

**Table 2:** List of South American, African and European populations used in population comparison analysis.

Code	Population	N	Reference
1	Manaus - Brazil, State of Amazon	42	this study
2	Ribeirão Preto - Brazil, State of São Paulo	65	this study
3	Santa Catarina - Brazil, State of Santa Catarina	109	Cainé *et al.*, 2005
4	Belém - Brazil, State of Pará	200	Palha *et al.*, 2007
5	Rio de Janeiro - Brazil, State of Rio de Janeiro	245	Goes *et al.*, 2005
6	São Paulo - Brazil, State of São Paulo	200	Gois *et al.*, 2007
7	Rio Grande Sul - Brazil, State of Rio Grande do Sul	203	Leite *et al.*, 2008
8	Rio de Janeiro - Brazil, State of Rio de Janeiro (African descendents)	135	Domingues *et al.*, 2007
9	Rio Grande do Sul - Brazil, Guarani and Kaingang (Amerindians)	42	Leite *et al.*, 2008
10	Argentina, northern region, Colla - (Amerindians)	48	Toscanini *et al.*, 2008
11	Argentina, northwestern region, Toba (Amerindians)	49	Toscanini *et al.*, 2008
12	Buenos Aires - Argentina	100	Sanchez-Diz *et al.*, 2008
13	Portugal, northern region	244	Sanchez-Diz *et al.*, 2008
14	Portugal, central region	100	Bento *et al.*, 2009
15	Portugal, southern region	100	Sanchez-Diz *et al.*, 2008
16	Spain	148	Martin *et al.*, 2004
17	Guinea Bissau	32	this study
18	Angola	48	this study
19	Mozambique	36	this study
20	Guinea Equatorial	101	Arroyo Pardo *et al.*, 2005
21	Cabinda - Angola	208	Beleza, 2005
22	Maputo - Mozambique,	112	Alves *et al.*, 2003
23	São Tome and Principe	103	Trovoada *et al.*, 2001
24	Cape Verde	47	Corte-Real *et al.*, 2000
