# Supplementary material for: Analysis of paternal lineages in Brazilian and African populations
Source: Genet Mol Biol. 2010 Sep 1;33(3):422–7. doi: 10.1590/S1415-47572010005000067 (PMC3036106; doi:10.1590/S1415-47572010005000067)
Supplement: Table S1 — Y chromosome haplotype distribution in the Manaus population sample (N = 42). [file gmb-33-3-422-suppl1.pdf]

Table S1. Y chromosome haplotype distribution in the Manaus population sample (N=42).

code	n	DYS19	DYS389I	DYS389II	DYS390	DYS391	DYS392	DYS393	DYS385	HG
M1	1	14	13	29	24	11	13	13	10,13	F*(xQ1a3a)-M213
M2	1	15	12	28	22	10	11	13	12,12	F*(xQ1a3a)-M213
M3	1	14	13	29	24	11	13	13	11,14	F*(xQ1a3a)-M213
M4	1	14	13	29	25	11	13	13	11,15	F*(xQ1a3a)-M213
M5	1	14	14	31	23	10	11	12	13,16	F*(xQ1a3a)-M213
M6	1	15	12	28	24	10	11	13	13,17	F*(xQ1a3a)-M213
M7	1	13	13	29	24	9	11	13	13,14	E - M35
M8	1	14	12	30	25	10	14	13	13,17	Q1a3a - M3
M9	1	14	12	28	23	10	11	13	13,14	F*(xQ1a3a)-M213
M10	1	14	12	28	24	12	13	13	11,14	F*(xQ1a3a)-M213
M11	1	14	13	31	22	10	11	12	13,16	F*(xQ1a3a)-M213
M12	1	14	13	29	25	10	13	13	11,15	F*(xQ1a3a)-M213
M13	1	13	13	30	24	10	11	13	16,18	E - M35
M14	1	13	14	30	23	10	14	13	14,16	Q1a3a - M3
M15	1	13	13	30	24	10	14	13	13,16	Q1a3a - M3
M16	1	15	13	29	22	10	13	12	11,15	F*(xQ1a3a)-M213
M17	1	15	13	28	24	10	13	13	12,13	F*(xQ1a3a)-M213
M18	1	14	13	29	23	10	11	12	14,16	F*(xQ1a3a)-M213
M19	1	13	13	29	25	9	11	13	13,14	E - M35
M20	1	15	13	29	23	11	14	13	11,13	F*(xQ1a3a)-M213
M21	1	14	13	29	23	11	13	13	11,14	F*(xQ1a3a)-M213
M22	1	14	13	28	25	11	13	13	11,14	F*(xQ1a3a)-M213
M23	1	16	13	28	23	10	11	13	12,12	F*(xQ1a3a)-M213
M24	1	13	13	31	25	10	14	13	14,14	Q1a3a - M3
M25	1	15	13	31	21	10	11	13	17,17	E - M2
M26	1	18	13	31	23	10	14	13	15,18	F*(xQ1a3a)-M213
M27	1	14	14	30	24	10	13	13	11,15	F*(xQ1a3a)-M213
M28	1	13	13	31	24	10	11	13	16,18	E - M35
M29	1	14	13	29	25	11	14	13	12,14	F*(xQ1a3a)-M213
M30	1	14	13	29	24	12	13	13	11,14	F*(xQ1a3a)-M213
M31	1	14	13	29	23	11	13	12	11,15	F*(xQ1a3a)-M213
M32	1	14	12	28	23	10	12	13	14,14	F*(xQ1a3a)-M213
M33	1	15	13	30	24	10	12	14	15,15	F*(xQ1a3a)-M213
M34	1	15	13	29	25	11	13	13	12,14	F*(xQ1a3a)-M213
M35	1	15	12	26	24	11	13	13	12,14	F*(xQ1a3a)-M213
M36	1	13	14	31	24	10	11	12	16,18	E - M35
M37	1	13	13	28	24	11	13	13	11,14	F*(xQ1a3a)-M213
M38	1	13	13	29	24	11	13	13	12,14	F*(xQ1a3a)-M213
M39	1	14	14	30	24	10	13	13	11,14	F*(xQ1a3a)-M213
M40	1	13	13	30	23	10	11	13	17,17	E - M35
M41	1	14	14	31	23	9	11	13	12,14	E - M35
M42	1	14	13	29	24	11	12	13	12,14	F*(xQ1a3a)-M213
