# Supplementary material for: Analysis of paternal lineages in Brazilian and African populations
Source: Genet Mol Biol. 2010 Sep 1;33(3):422–7. doi: 10.1590/S1415-47572010005000067 (PMC3036106; doi:10.1590/S1415-47572010005000067)
Supplement: Table S2 — Y chromosome haplotype distribution in the Ribeirão Preto population sample (N = 65). [file gmb-33-3-422-suppl2.pdf]

Table S2. Y chromosome haplotype distribution in the Ribeirão Preto population sample (N=65).

code	n	DYS19	DYS389I	DYS389II	DYS390	DYS391	DYS392	DYS393	DYS385	HG
RP1	4	14	13	29	24	11	13	13	11-14	F*(xQ1a3a)-M213
RP2	1	13	14	30	24	9	11	13	13-14	E1b1b1 - M35
RP3	1	15	12	28	25	11	11	12	18-18	F*(xQ1a3a)-M213
RP4	1	15	13	29	24	11	13	13	11-13	F*(xQ1a3a)-M213
RP5	2	14	13	29	24	11	13	13	11-11	F*(xQ1a3a)-M213
RP6	3	14	13	30	24	10	13	13	11-14	F*(xQ1a3a)-M213
RP7	1	15	13	30	24	10	12	15	14-15	F*(xQ1a3a)-M213
RP8	1	14	13	30	24	10	11	12	13-17	F*(xQ1a3a)-M213
RP9	1	14	13	29	23	10	13	14	11-14	F*(xQ1a3a)-M213
RP10	1	14	13	28	24	11	13	13	11-13	F*(xQ1a3a)-M213
RP11	1	15	13	29	22	11	13	12	11-14	F*(xQ1a3a)-M213
RP12	1	15	13	29	24	11	13	13	11-14	F*(xQ1a3a)-M213
RP13	1	14	13	30	24	10	13	13	11-11	F*(xQ1a3a)-M213
RP14	1	15	12	28	24	10	14	13	14-16	F*(xQ1a3a)-M213
RP15	1	15	13	29	24	10	13	13	12-14	F*(xQ1a3a)-M213
RP16	1	15	13	30	24	9	11	13	17-17	E1b1b1 - M35
RP17	1	17	13	31	25	10	12	14	12-15	F*(xQ1a3a)-M213
RP18	1	16	13	30	25	11	11	13	11-14	F*(xQ1a3a)-M213
RP19	2	14	14	30	24	10	13	13	11-14	F*(xQ1a3a)-M213
RP20	1	14	13	31	24	11	11	15	15-16	E1b1b1 - M35
RP21	1	13	13	30	24	9	11	13	17-17	E1b1b1 - M35
RP22	1	14	13	30	23	10	11	12	13-18	F*(xQ1a3a)-M213
RP23	1	14	13	29	24	10	13	13	13-13	F*(xQ1a3a)-M213
RP24	1	14	14	30	25	10	13	14	11-14	F*(xQ1a3a)-M213
RP25	1	15	13	29	24	10	11	12	14-17	F*(xQ1a3a)-M213
RP26	1	14	13	30	24	10	13	14	14-14	F*(xQ1a3a)-M213
RP27	1	15	12	30	22	10	11	14	14-15	F*(xQ1a3a)-M213
RP28	1	14	13	29	25	10	13	13	11-14	F*(xQ1a3a)-M213
RP29	1	15	12	28	24	10	11	12	13-18	F*(xQ1a3a)-M213
RP30	1	14	13	29	24	10	11	12	14-15	F*(xQ1a3a)-M213
RP31	1	14	13	31	24	10	13	13	11-14	F*(xQ1a3a)-M213
RP32	1	15	13	30	24	10	13	13	11-14	F*(xQ1a3a)-M213
RP33	1	14	14	30	24	10	11	12	13-19	F*(xQ1a3a)-M213
RP34	1	14	14	31	24	11	13	15	11-14	F*(xQ1a3a)-M213
RP35	1	15	13	30	21	10	12	13	11-12	A*
RP36	1	14	12	29	23	10	15	13	13-18	Q1a3a - M3
RP37	1	16	13	30	25	10	11	13	11-13	F*(xQ1a3a)-M213
RP38	1	15	13	29	23	11	13	13	11-14	F*(xQ1a3a)-M213
RP39	1	15	13	30	21	11	11	13	17-19	E1b1a - M2
RP40	1	17	14	30	25	10	11	13	14-17	E1b1b1 - M35
RP41	1	14	14	30	24	10	11	12	13-17	F*(xQ1a3a)-M213
RP42	1	14	14	30	24	10	11	13	13-15	F*(xQ1a3a)-M213
RP43	1	14	12	29	22	10	11	13	14-15	F*(xQ1a3a)-M213
RP44	1	14	10	26	24	11	14	13	12-14	F*(xQ1a3a)-M213
RP45	1	15	13	29	24	11	13	13	9-14	F*(xQ1a3a)-M213
RP46	1	14	13	28	24	11	13	13	11-14	F*(xQ1a3a)-M213
RP47	1	15	12	28	24	10	11	12	15-17	F*(xQ1a3a)-M213
RP48	1	13	14	31	24	10	11	13	13-15	Y* (A,E1b1a-b,F*) - SRY10831.1
RP49	1	14	13	29	24	10	13	13	11-14	F*(xQ1a3a)-M213
RP50	1	15	12	28	22	10	11	13	13-14	F*(xQ1a3a)-M213

Table S2. Y chromosome haplotype distribution in the Ribeirão Preto population sample (N=65) (Cont.).

RP51	1	14	12	28	23	10	12	13	11-14	F*(xQ1a3a)-M213
RP52	1	15	12	27	25	10	14	13	13-16	F*(xQ1a3a)-M213
RP53	1	14	14	30	24	11	13	13	10-13	F*(xQ1a3a)-M213
RP54	1	16	13	29	23	11	12	14	15-16	F*(xQ1a3a)-M213
RP55	1	13	14	31	24	11	11	12	16-18	E1b1b1 - M35
RP56	1	15	12	29	25	10	11	12	13-17	F*(xQ1a3a)-M213
RP57	1	14	12	28	25	10	13	13	11-14	F*(xQ1a3a)-M213
RP58	1	14-15	12	28	22	10	11	14	12-13	F*(xQ1a3a)-M213
