# Supplementary material for: Analysis of paternal lineages in Brazilian and African populations
Source: Genet Mol Biol. 2010 Sep 1;33(3):422–7. doi: 10.1590/S1415-47572010005000067 (PMC3036106; doi:10.1590/S1415-47572010005000067)
Supplement: Table S3 — Y chromosome haplotype distribution in the Guinea Bissau population sample (N = 32). [file gmb-33-3-422-suppl3.pdf]

Table S3. Y chromosome haplotype distribution in the Guinea Bissau population sample (N=32).

code	n	DYS19	DYS389I	DYS389II	DYS390	DYS391	DYS392	DYS393	DYS385
G1	1	13	13	31	24	10	12	14	16,17
G2	1	14	13	30	23	10	11	14	11,14
G3	1	15	12	29	22	11	11	13	14,14
G4	1	15	13	30	21	10	11	14	14,14
G5	1	15	13	30	21	10	11	15	16,16
G6	1	15	13	30	22	11	11	13	15,15
G7	1	15	13	31	21	10	11	13	16,17
G8	1	15	13	31	21	10	11	13	16,19
G9	1	15	13	32	21	10	11	14	15,16
G10	1	15	13	32	21	11	11	14	15,15
G11	1	15	14	30	21	10	11	14	16,18
G12	2	15	14	31	21	10	11	14	14,14
G13	1	15	14	32	23	11	11	13	17,17
G14	1	15	15	32	22	10	11	14	14,16
G15	1	16	12	30	22	10	11	13	14,16
G16	1	16	12	30	22	11	11	13	15,17
G17	1	16	13	29	21	10	11	14	15,15
G18	1	16	13	30	21	10	11	13	16,17
G19	1	16	13	30	21	10	11	14	17,17
G20	1	16	13	30	21	10	12	15	16,17
G21	1	16	14	30	21	10	11	14	17,17
G22	1	16	14	30	22	10	11	13	17,17
G23	1	17	12	29	22	10	11	13	14,17
G24	1	17	13	30	20	10	11	14	15,17
G25	1	17	13	30	21	10	11	14	16,18
G26	1	17	13	31	20	11	11	15	16,16
G27	1	17	14	31	22	10	11	13	15,16
G28	3	17	14	31	22	10	11	13	16,17
G29	1	17	14	31	22	10	11	13	17,17
