# Supplementary material for: Analysis of paternal lineages in Brazilian and African populations
Source: Genet Mol Biol. 2010 Sep 1;33(3):422–7. doi: 10.1590/S1415-47572010005000067 (PMC3036106; doi:10.1590/S1415-47572010005000067)
Supplement: Table S4 — Y chromosome haplotype distribution in the Angola population sample (N = 48). [file gmb-33-3-422-suppl4.pdf]

Table S4. Y chromosome haplotype distribution in the Angola population sample (N=48).

code	n	DYS19	DYS389I	DYS389II	DYS390	DYS391	DYS392	DYS393	DYS385
A1	2	14	12	28	25	10	11	13	14,20
A2	1	14	12	28	25	11	11	13	14,20
A3	1	14	12	28	26	11	11	13	14,19
A4	1	14	13	30	21	10	11	14	14,19
A5	2	15	12	28	22	10	11	14	12,13
A6	1	15	12	29	21	10	11	14	16,17
A7	1	15	13	30	21	10	11	13	15,18
A8	1	15	13	30	21	10	11	13	16,17
A9	1	15	13	30	21	10	11	13	16,18
A10	2	15	13	30	21	10	11	13	17,17
A11	1	15	13	30	21	10	11	14	12,16
A12	1	15	13	30	21	10	11	14	14,18
A13	1	15	13	30	21	10	11	14	15,16
A14	2	15	13	30	21	11	11	13	16,17
A15	1	15	13	30	21	11	11	14	16,17
A16	1	15	13	31	21	10	11	13	15,15
A17	1	15	13	31	21	10	11	13	15,17
A18	1	15	13	31	21	10	11	13	16,16
A19	2	15	13	31	21	10	11	13	16,17
A20	1	15	13	31	21	10	11	13	17,17
A21	1	15	13	31	21	10	11	13	17,18
A22	2	15	13	31	21	11	11	13	16,17
A23	1	15	13	31	21	12	11	13	16,17
A24	1	15	14	30	24	12	13	13	16,17
A25	1	15	14	31	21	10	11	13	15,19
A26	1	15	14	32	21	11	11	13	15,17
A27	1	15	14	32	24	10	11	13	11,11
A28	1	15	14	33	21	10	11	13	15,20
A29	1	16	12	30	24	10	11	13	11,11
A30	1	16	13	29	21	10	11	15	16,16
A31	1	16	13	30	21	10	11	14	15,17
A32	1	16	13	30	21	10	11	14	18,18
A33	1	16	13	30	21	11	11	13	17,17
A34	1	16	13	30	21	11	11	14	16,18
A35	1	16	13	31	21	10	11	15	16,17
A36	1	16	13	31	21	11	11	13	17,17
A37	1	16	14	31	21	10	11	15	16,20
A38	1	16	14	32	25	10	11	13	11,11
A39	1	17	13	30	21	11	11	15	17,19
A40	1	17	13	30	21	11	11	15	18,19
A41	1	17	14	31	21	10	11	15	16,18
A42	1	17	14	32	21	10	11	13	17,18
