# Supplementary material for: Analysis of paternal lineages in Brazilian and African populations
Source: Genet Mol Biol. 2010 Sep 1;33(3):422–7. doi: 10.1590/S1415-47572010005000067 (PMC3036106; doi:10.1590/S1415-47572010005000067)
Supplement: Table S5 — Y chromosome haplotype distribution in the Mozambique population sample (N = 36). [file gmb-33-3-422-suppl5.pdf]

Table S5. Y chromosome haplotype distribution in the Mozambique population sample (N=36).

code	n	DYS19	DYS389I	DYS389II	DYS390	DYS391	DYS392	DYS393	DYS385
Mo1	1	12	13	30	21	10	11	14	17,18
Mo2	1	13	11	29	23	11	11	14	16,16
Mo3	1	14	12	28	24	11	11	13	13,19
Mo4	2	14	12	28	24	11	11	13	14,19
Mo5	1	14	12	28	27	10	11	13	13,19
Mo6	2	14	13	30	21	11	11	14	16,16
Mo7	1	14	13	31	23	10	10	14	14,17
Mo8	1	15	12	26	20	10	11	13	15,17
Mo9	1	15	12	30	21	10	11	13	17,18
Mo10	1	15	13	30	21	10	11	13	16,17
Mo11	1	15	13	30	21	10	11	14	16,16
Mo12	1	15	13	30	21	10	11	14	17,17
Mo13	1	15	13	30	21	11	11	13	16,17
Mo14	1	15	13	30	21	12	11	14	16,17
Mo15	1	15	13	31	21	10	11	13	16,16
Mo16	1	15	13	31	21	10	11	13	16,18
Mo17	1	15	13	31	21	10	11	14	15,20
Mo18	1	15	14	28	25	11	11	13	10,15
Mo19	1	15	14	31	21	10	11	13	15,17
Mo20	1	15	14	31	21	11	11	14	15,20
Mo21	1	15	14	32	24	10	11	13	11,11
Mo22	1	15	14	32	24	10	12	15	11,12
Mo23	1	15	14	32	24	11	11	13	11,11
Mo24	1	15	14	33	24	10	13	13	11,12
Mo25	1	16	12	28	25	10	11	13	11,14
Mo26	1	16	13	30	20	10	11	16	16,18
Mo27	1	16	14	31	21	10	11	14	17,18
Mo28	1	16	14	32	24	11	11	13	10,11
Mo29	1	17	13	30	21	10	11	14	16,16
Mo30	1	17	13	30	21	10	11	14	16,17
Mo31	1	17	13	30	21	10	11	14	17,17
Mo32	1	17	13	30	25	10	11	15	17,18
Mo33	1	17	13	31	21	10	11	14	17,18
Mo34	1	17	15	32	21	10	11	14	15,19
